# Supplementary material for: Efficacy and Safety of the Addition of Internal Mammary Irradiation to Standard Adjuvant Radiation in Early-Stage Breast Cancer: A Systematic Review and Meta-Analysis
Source: Curr Oncol. 2022 Sep 17;29(9):6657–73. doi: 10.3390/curroncol29090523 (PMC9497563; doi:10.3390/curroncol29090523)
Supplement: Supplementary file 1 [file curroncol-29-00523-s001.zip › curroncol-1878631-supplementary.pdf]

Current Oncology

MDPI

Systematic Review

Efficacy and Safety of the Addition of Internal Mammary Irradiation to Standard Adjuvant Radiation in Early-Stage Breast Cancer: A Systematic Review and Meta-Analysis

Yasmin Korzets, Dina Levitas, Ahuva Grubstein, Benjamin W. Corn, Eitan Amir and Hadar Goldvaser

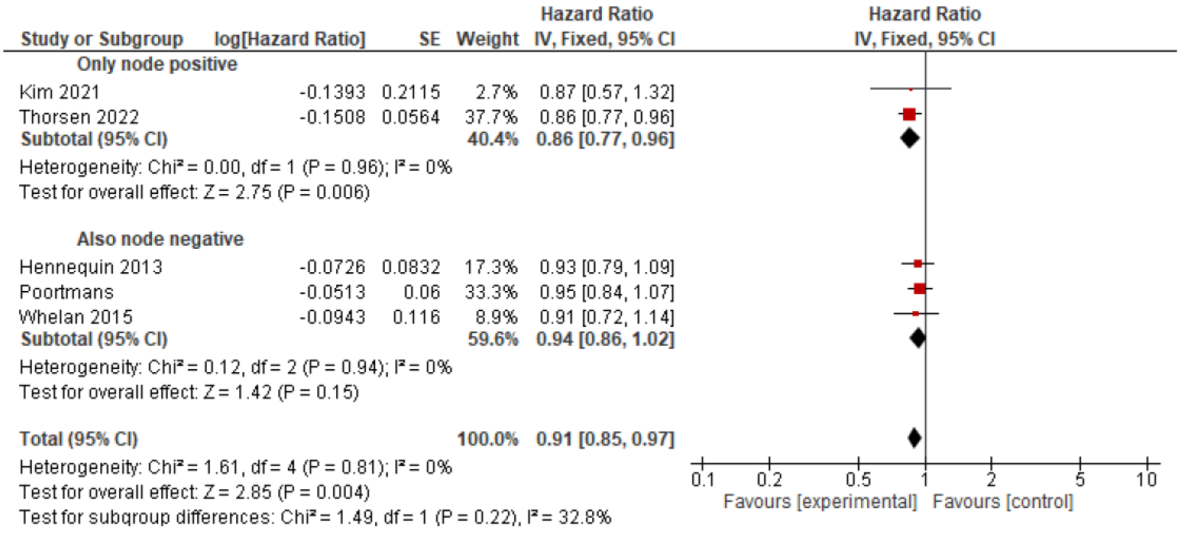

A

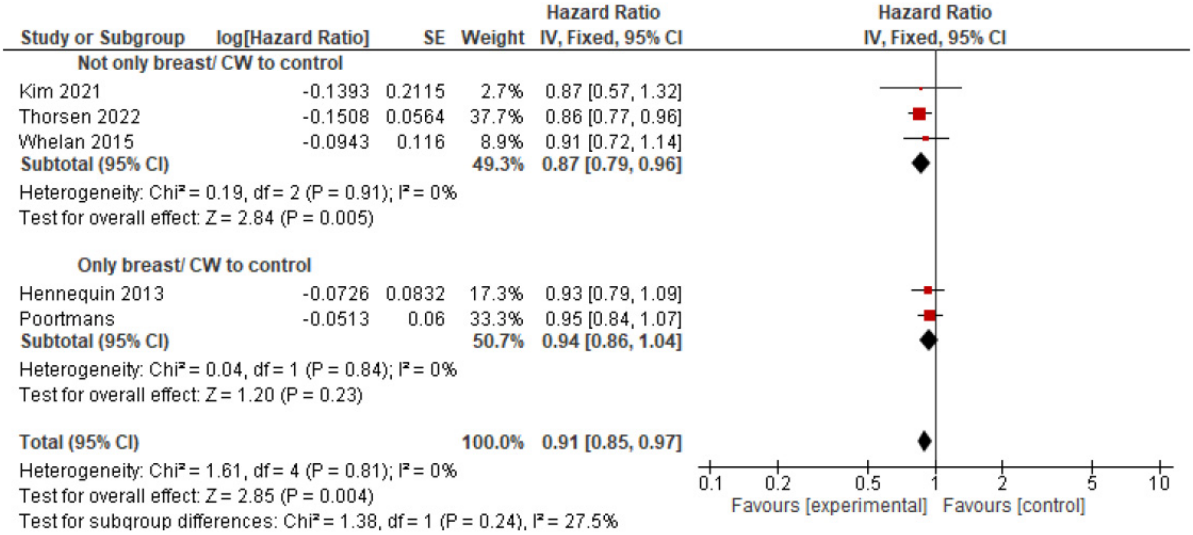

B

Figure S1. Forest plots for overall survival by study design, hazard ratio for: A: Overall survival by inclusion of node negative disease- inclusion of only node positive disease compared to inclusion of both node negative and node positive disease. B: Overall survival by extend of treatment to the control group- radiation to the control group comprised breast/ chest wall only compared to breast/ chest wall and other regional lymph nodes). References: Hennequin 2013 [13], Kim et al., 2021 [24], Poortmans 2020 [12], Thorsen 2022 [15], Whelan 2015 [11].
